# How did previously obese children and adolescents behave during the SARS-CoV-2 pandemic in relation to weight gain?

**DOI:** 10.1590/1984-0462/2025/43/2024058

**Published:** 2024-11-29

**Authors:** Mirella Menaque da Paz, Fábio de Freitas, Mariana Renata Zago, Mariana Porto Zambon, Maria Ângela Reis de Góes Monteiro Antônio

**Affiliations:** a Universidade Estadual de Campinas, Campinas, SP, Brazil.

**Keywords:** Children, Adolescents, Pediatric obesity, Weight gain, Pandemic, Crianças, Adolescentes, Obesidade pediátrica, Ganho de peso, Pandemia

## Abstract

**Objective:**

The objective of this study was to analyze the implications of social contingency measures and interruption of outpatient follow-up on weight gain in children and adolescents with a previous diagnosis of obesity.

**Methods:**

This is an observational study with data from electronic medical records of children and adolescents followed up at a specialized outpatient clinic from 2019 to 2023. Weight gain, height, BMI variation, BMI z-score, laboratory tests, and associated comorbidities were analyzed. The data were computed and analyzed using the Statistical Package for the Social Sciences (SPSS), and the results were considered statistically significant when p<0.05.

**Results:**

There was a weight gain of approximately 17.66% in the total set of participants, corresponding to a median increase of 14 kg. When analyzing between genders, we observed an approximate increase of 21.38% in body weight for men, while for women, it was 21.45%.

**Conclusions:**

The COVID-19 pandemic has led to significant weight gain among previously obese children and adolescents in follow-up at a specialized outpatient clinic.

## RESUMO

**Objetivo:**

Analisar as implicações das medidas de contingenciamento social e interrupção do acompanhamento ambulatorial sobre o ganho de peso de crianças e adolescentes com diagnóstico prévio de obesidade.

**Métodos:**

Estudo observacional com dados proveniente de prontuários eletrônicos de crianças e adolescentes acompanhadas no Ambulatório de Obesidade na Criança e no Adolescente do Hospital de Clínicas da Universidade Estadual de Campinas, no período de 2019 a 2023. Analisaram-se ganho ponderal, altura, variação do índice de massa corporal (IMC), escore Z de IMC, exames laboratoriais e comorbidade associadas. Os dados foram computados e analisados pelo programa *StatisticalPackage for the Social Sciences* (SPSS). Os resultados foram considerados estatisticamente significativos quando p<0,05.

**Resultados:**

Constatou-se aumento ponderal de aproximadamente 17,6% no conjunto total de participantes, correspondendo ao acréscimo mediano de 14 kg. Ao analisarmos os gêneros, observamos elevação aproximada de 21,38% no peso corporal do sexo masculino, enquanto a do sexo feminino foi de 21,4%.

**Conclusões:**

A pandemia de COVID-19 levou a ganho peso significante entre crianças e adolescentes previamente obesos, que estavam em seguimento em ambulatório especializado.

## INTRODUCTION

The increase in obesity levels in children and adolescents has been a cause for concern among health agencies globally.^1^ In Brazil, statistics from 2019 show that 14.96% of children aged 5–10 years are overweight, 8.22% are obese, and 4.97% face severe obesity. Among adolescents, 18.25% are overweight, 7.91% are obese, and 1.8% face severe obesity.^2^ This increase in obesity in the pediatric population can have physical, mental, and social consequences, with comorbidities that affect the short-, medium-, and long-term.^3^

In January 2020, the World Health Organization (WHO) declared the epidemic of the new coronavirus (SARS-CoV-2) a public health emergency of international concern.^4^ To reduce the spread of the virus, many countries, including Brazil, have implemented confinement measures. However, such measures had significant impacts on the lifestyle, physical, and mental health of children and adolescents.^5,6^

With the closure of educational institutions — one of the social distancing measures — activities associated with active commuting to school, physical education classes with the mandatory practice of sports, games, and/or games during breaks were abandoned, so that time out of school was associated with the interruption of a more active routine.^5,7,8^ The set of restrictive measures adopted reduced the time of physical activity and encouraged sedentary behavior.^9^

Such changes made it possible to experience a feeling of frustration, boredom, and, above all, anxiety,^10-13^ which would act as a risk factor for the worsening of the childhood obesity pandemic due to eating behaviors that arise in response to new stressors.^11,14^ Maintaining a healthy routine has become an arduous task in this context.

Thus, the COVID-19 pandemic, which in itself is a major global public health challenge, may aggravate childhood obesity, which affects around 158 million children and adolescents worldwide.^15^

In this context, the aim of this study was to evaluate how children and adolescents previously diagnosed with obesity behaved during the SARS-CoV-2 pandemic about weight gain.

## METHOD

This study was approved by the Research Ethics Committee, following the norms established by Resolution 466/2012 of the National Health Council. All participants and their guardians who agreed to participate signed the free and informed consent form. The data use commitment term was used in case of loss of follow-up and death.

The Centers for Disease Control and Prevention (CDC) curves were used to diagnose obesity in children and adolescents of both sexes aged between 2 and 18 years.^16^ These individuals were followed up at the Child and Adolescent Obesity Outpatient Clinic from 2019 to 2023.

This observational research was based on the analysis of data series from electronic medical records, where the consultations performed at the multidisciplinary outpatient clinic are recorded.

As an inclusion criterion, we considered the last pre-pandemic consultation, covering the period between January 2019 and March 2020, adopted as a baseline, and the first post-pandemic consultation, which was carried out between November 2020 and May 2023, was defined for comparison. This time interval encompasses the period of greater social isolation during the SARS-CoV-2 pandemic, as recommended by regulatory bodies in Brazil.

All data were collected at baseline and at the first post-pandemic visit. The following data were collected: weight, height, and waist circumference (WC). Body mass index (BMI) was calculated as body weight in kilograms divided by the square of height in meters (BMI=weight (kg)/height^2^ (m)). The BMI Z-score was calculated according to the WHO reference using the AnthroPlus calculator (version 1.0.4, WHO).^17^

The blood pressure (BP) was assessed by a pediatrician using the auscultatory technique with a mercury spine sphygmomanometer and a cuff with a rubber bag of appropriate size for the arm circumference.^18^

The laboratory tests included were total cholesterol, high-density lipoprotein (HDL), low-density lipoprotein (LDL), triglycerides (TG), and fasting glucose, which are collected annually from patients who follow up at the outpatient clinic. The laboratory tests were performed in the Clinical Pathology Laboratory using standardized techniques.

The diagnoses related to the other clinical conditions were checked and noted to identify the most prevalent comorbidities between pre- and post-pandemic consultations for further analyses, highlighting among them atopy conditions such as asthma and allergic rhinitis, allergic conjunctivitis, depressive anxiety disorders, systemic arterial hypertension, dyslipidemia, diabetes mellitus, and hepatic steatosis.

Statistical analysis was performed using IBM SPSS (version 28.0, IBM, New York, USA). The Shapiro-Wilk test was applied to assess the normality of data distribution. The descriptive characteristics were presented as mean and standard deviation (parametric distribution) and median and interquartile range (non-parametric distribution). Differences between genders were analyzed using the independent t-test and the Mann-Whitney test for normal and asymmetric data, respectively. Quantile regression analysis (adjusted for age) was used to analyze longitudinal changes over time (pre- and post-pandemic) and between sexes in body composition and laboratory tests. 95% confidence intervals were established. The analyses of the most prevalent comorbidities were performed after grouping by categories using the set of multiple responses and presented in absolute and relative frequencies.

A p-value ≤ 0.05 was considered statistically significant.

## RESULTS

A total of 73 electronic medical records were analyzed, most of whom were male (58.9%). The mean age was 10.24 (±3.54), the median BMI was 28.10 (interquartile range, IQR: 7.65) kg/m^2^, and the BMI Z-score was 3.20 (IQR: 1.19).

Overall, the data showed a median weight of 64.00 (IQR: 38.43) kg, height of 148.50 (IQR: 33.75) cm, and WC of 92.48 (IQR: 7.64) cm, with no statistical difference between genders. A statistically significant difference was observed at baseline in BMI z-score (*p*=0.031) between the sexes (Table 1).

**Table 1:** Characteristics of the sample.

	Total (n=73)	Male (n=43)	Female (n=30)	p-value
Age (years)	10.24±3.54	9.99±3.71	10.61±3.31	0.465^a^
Weight (kg)	64.00 (38.40)	64.39±27.75	65.89±30.47	0.828^a^
Height (cm)	148.50 (33.75)	148.50 (39.40)	148.45 (23.00)	0.711^b^
BMI (kg/m^2^)	28.10 (7.65)	28.10 (7.00)	28.20 (8.58)	0.699^b^
BMI Z-score	3.20 (1.19)	3.30 (1.68)	2.89 (1.05)	0.031^b^
WC (cm)	92.48 (7.64)	92.48 (6.50)	92.48 (10.49)	0.230^b^
Pubertal stages (%)				
Pre-school	7 (9.6)			
School	12 (16.4)			
Pre-pubertal	14 (19.2)			
Pubertal	33 (45.2)			
Post-pubertal	7 (9.6)			

Mean±SD, standard deviation; BMI, body mass index; WC, waist circumference;

^a^ and

^b^, Differences between groups analyzed with the independent t-test and the Mann-Whitney U test, respectively; Median, interquartile range.

According to mixed-effects quantile regression analysis, time effects were observed in which there was an increase in weight (β=14.76; 95%CI 11.89–17.62; p<0.001) kg, BMI (β=3.31; 95%CI 2.44–4.18; p<0.001) kg/m^2^, BMI Z-score (β=0.20; 95%CI 0.10–0.41; p=0.047), and WC (β=9.64; 95%CI 8.32–10.97; p<0.001) cm (Table 2). No significant differences were observed between the sexes (Table 2).

**Table 2:** Comparative analysis of pre- and post-pandemic participants of anthropometry, body composition, and laboratory tests.

	Total (n=73)		Male (n=43)		Female (n=30)		Time*	Sex*
	Baseline	After	Baseline	After	Baseline	After	β (95%CI)	β (95%CI)
Weight (kg)	64.00 (38.43)	75.25 (34.88)	65.00 (42.00)	78.85 (37.25)	61.08 (34.74)	74.20 (38.25)	**14.76** **(11.89,17.62)**	-3.93 (-11.70, 3.83)
Height (cm)	148.50 (33.75)	158.00 (27.05)	148.50 (39.40)	160.00 (34.00)	148.45 (23.00)	156.65 (15.50)	**8.27** **(6.64, 9.91)**	**-5.92** **(-11.27, -.0.56)**
BMI (kg/m^2^)	28.10 (7.65)	31.10 (8.10)	28.10 (7.00)	31.90 (7.10)	28.20 (8.58)	30.00 (14.03)	**3.31** **(2.44, 4.18)**	0.47 (-1.97. 2.93)
BMI Z-score	3.20 (1.19)	3.29 (1.65)	3.30 (1.68)	3.48 (1.67)	2.89 (1.05)	3.29 (1.37)	**0.20** **(0.00, 0.41)**	-0.51 (-1.05, 0.02)
WC (cm)	92.48 (764)	102.20 (9.50)	92.48 (6.50)	102.20 (5.53)	92.48 (10.49)	102.20 (19.78)	**9.64** **(8.32, 10.97)**	-4.71 (-9.95, 0.53)
Laboratory tests								
Cholesterol (mg/dL)	151.08 (23.26)	156.27 (29.50)	151.08 (22.98)	156.27 (34.39)	151.96 (26.79)	153.50 (28.50)	**3.02** **(0.12, 5.93)**	-5.57 (-15.78, 4.63)
HDL (mg/dL)	41.35 (5.28)	39.00 (5.98)	41.06 (6.29)	39.00 (5.00)	41.42 (5.36)	39.83 (6.25)	**-1.69** **(-2.75, -0.63)**	7.66 (-1.09, 2.63)
LDL (mg/dL)	91.17 (17.59)	96.48 (19.61)	91..17 (21.82)	98.00 (28.75)	91.17 (22.63)	93.86 (16.73)	**5.17** **(2.81, 7.54)**	-2.65 (-10.30, 4.98)
TG (mg/dL)	103.26 (43.32)	112.00 (56.97)	99.57 (44.77)	116.53 (70.67)	104.13 (36.88)	109.50 (46.86)	**10.69** **(0.71, 21.31)**	-3.77 (-25.42, 17.86)
Glucose (mg/dL)	82.84 (3.34)	82.59 (7.50)	82.96 (3.59)	83.00 (8.00)	82.84 (3.45)	82.59 (4.28)	-0.01 (-1.64, 1.60)	-0.63 (-2.72, 1.45)

Median, interquartile range; BMI, body mass index; zBMI, BMI z-score; WC, waist circumference; HDL, high-density lipoprotein; LDL, low-density lipoprotein; TG, triglycerides.

*Quantile regression for mixed models; β in bold indicates a significant interaction (p≤0.05); β, the expected value of the median (time – pre- and post-pandemic); sex (girls as a reference category); βs standardized and adjusted for age.

An increase in total cholesterol levels (β=3.02; 95%CI 0.12–5.93; p=0.0441), reduction in HDL (β=1.69; 95%CI -2.75 to -0.63; p=0.023) mg/dL, increased LDL (β=5.17; 95%CI 2.81–7.54; p<0.001) mg/dL, and TG (β=10.69; 95%CI 0.07–21.31; p=0.048) mg/dL. No significant effect was observed for blood glucose (β=-0.01; 95%CI -1.64 to 1.60; p=0.981) mg/dL. No significant interactions were observed between the sexes (Table 2).

Before the pandemic, the most common diagnoses among boys included asthma (22.4%), systemic arterial hypertension (10.3%), and fatty liver disease (3.4%). Among girls, the same conditions also stood out: asthma (21.3%), systemic arterial hypertension (10.6%), and anxiety (4.3%).

After the impact of the pandemic, asthma remained the most prevalent comorbidity in both sexes, but with slightly reduced rates (20.5% among boys and 24.1% among girls). SAH also maintained its relevance with a prevalence of 9.6% and 13.0% among boys and girls, respectively (Table 3).

**Table 3:** The most prevalent comorbidities between pre- and post-pandemic consultations.

Comorbidities	Male		Female	
	Baseline	After	Baseline	After
n	58	47	73	54
Asthma	13 (22.4)	15 (20.5)	10 (21.3)	13 (24.1)
Hepatic steatosis	2 (3.4)	4 (5.5)	1 (2.1)	2 (3.7)
SAH	6 (10.3)	7 (9.6)	5 (10.6)	7 (13.0)
Anxiety	1 (1.7)	4 (5.5)	2 (4.3)	3 (5.6)
Depression	1 (1.7)	-	1 (2.1)	1 (1.9)
Hypertriglyceridemia	1 (1.7)	3 (4.1)	1 (2.1)	1 (1.9)

Data presented as n (%); n, samples; SAH, systemic arterial hypertension.

## DISCUSSION

The aim of this study was to evaluate the impact of the SARS-CoV-2 pandemic on children and adolescents previously diagnosed with obesity, followed up in a multidisciplinary outpatient clinic. The results showed a significant increase in weight, BMI, BMI z-score, and WC. In addition, laboratory tests revealed elevations in total and LDL cholesterol levels, accompanied by a reduction in HDL. These findings indicate that this population, deprived of social interaction and daily activities, faced unfavorable conditions during isolation. School closures, restrictions in public areas, and stay-at-home guidelines have had a negative impact on body composition,^19^ observed with the increase in BMI levels and BMI Z-score. For children and adolescents with obesity, every 10% increase in BMI above the 95th percentile is equivalent to an average increase of 2.1–2.8 kg/m^2^ with possible clinically relevant impacts.^20^

The results showed a weight gain of approximately 17.6% in the body weight of the participants, corresponding to a median increase of approximately 14 kg. When analyzing between genders, we observed an approximate increase of 21.4% in body weight for men, while for women, it was 21.4%. The increase found in our study indicated a significant percentage of weight gain when compared to national statistics. In Brazil, during the period that encompassed the COVID-19 pandemic, the number of overweight children in the country grew by 6.1%, with an increase among adolescents of 17.2%.^21^

The metabolic variations observed in our study can be attributed to the impacts resulting from the confinement measures implemented during the COVID-19 pandemic based on the modification of the routine.^22,23^ As noted by Pietrobelli et al., only 3 weeks after the start of isolation, there was an increase in screen time, poor diet, and irregular sleep,^24^ and a significant reduction in both the duration and intensity of active body movements, resulting in a significant increase in time devoted to sedentary activities.^25^ This obesogenic environment experienced by children and adolescents may have contributed to a decrease in physical fitness, resulting in lower HDL levels and increased LDL.^26^

We observed an upward trend in the number of systemic arterial hypertension cases, representing approximately 26.9% of the cases. In addition, there was a propensity for an increase in the prevalence of fatty liver between pre- and post-pandemic consultations, showing that significant weight gain may be related.^27^

We also observed an increase in diagnoses of anxiety-depressive disorders in children, especially when compared to normative values before the pandemic. Our results, based on a comprehensive epidemiological analysis, are in agreement with previous findings indicating that children with obesity exhibited higher anxiety scores even before the direct onset of the pandemic’s impacts.^15,28^ The global health crisis has exacerbated these symptoms, leading to a 25% increase in the global prevalence of anxiety and depression in the first year of the pandemic.^29^ The increase in adiposity is associated with a series of psychological problems, which can have a considerable impact on the quality of life of this population, who suffer from excess weight and may have more symptoms of anxiety and depression.^30^ The reduction of outdoor activities and social interaction during periods of stricter isolation measures has been associated with these results.^12^

The main limitation of our study was the fact that we used electronic medical records as a data source. The lack of uniformity in clinical records made it difficult to include a larger number of cases in the analysis. This limitation may affect the generalization of our results to the general population and should be considered when interpreting the study findings. While we recognize the importance of sample size calculations, particularly in clinical trials, it is important to consider the inherent limitations of conducting research in pediatric populations. Due to the nature of the intervention and the specific constraints of this study, we chose to include all available participants within the established criteria. This approach was necessary to ensure the feasibility of the study, as the availability of fit pediatric patients is limited. However, we seek to mitigate any potential bias by using appropriate statistical methods and ensuring the representativeness of the sample within the context of the study.

On the contrary, the study showed the relationship between social isolation and the worsening of obesity and comorbidities in children and adolescents previously diagnosed with obesity. These results underscore concern about the future consequences of this unhealthy weight gain in childhood, both in the short- and long-term. In conclusion, our study contributed by showing that the COVID-19 pandemic led to significant weight gain among children and adolescents already followed up in a specialized outpatient clinic with a previous diagnosis of obesity. A higher incidence of comorbidities associated with excess weight, such as hypertension and fatty liver, was observed.

## Data Availability

The database that originated the article is available with the corresponding author.
